# Synthesis and crystal structure of di-μ-chlorido-bis­[bis(2,6-di­methyl­pyrazine)­copper(I)] and di-μ-bromido-bis[bis(2,6-di­methyl­pyrazine)copper(I)]

**DOI:** 10.1107/S2056989026004330

**Published:** 2026-05-07

**Authors:** Christian Näther

**Affiliations:** a Institut für Anorganische Chemie, Universität Kiel, Max-Eyth.-Str. 2, 24118 Kiel, Germany; University of Neuchâtel, Switzerland

**Keywords:** dinuclear complex, coordination compounds, copper(I) chloride, copper(I) bromide, 2,6-di­methyl­pyrazine, crystal structure

## Abstract

Crystals of [Cu_2_Cl_2_(C_6_H_8_N_2_)_4_] (**1**) and of [Cu_2_Br_2_(C_6_H_8_N_2_)_4_] (**2**), C_6_H_8_N_2_ = 2,6-di­methyl­pyrazine, were obtained from the reaction of CuCl or CuBr with 2,6-di­methyl­pyrazine in aceto­nitrile under solvothermal conditions. Both compounds are isotypic. The asymmetric unit of each compound consists of one Cu cation, one halide anion and one 2,6-di­methyl­pyrazine ligand in general positions. The copper cations are tetra­hedrally coordinated by two μ-1,1 bridging halide anions and two terminal 2,6-di­methyl­pyrazine ligands. Two copper cations are linked by the two halide anions *via* common edges into discrete dinuclear complexes that are located on centers of inversion. The discrete complexes are linked by inter­molecular C—H⋯*X* (*X* = Cl, Br) hydrogen bonding into chains that propagate along the *a*-axis direction. Between these chains, additional C–H⋯*X* inter­actions are observed, which might be stronger for compound **1**.

## Abstract 2

The crystal structures of [(CuCl)_2_(C_6_H_8_N_2_)_4_] (**1**) and [(CuBr)_2_(C_6_H_8_N_2_)_4_] (**2**) are isotypic and consists of dinuclear complexes, in which each copper cation is tetra­hedrally coordinated by two μ-1,1 bridging halide anions and two terminally coordinated 2,6-di­methyl­pyrazine ligands.

## 1. Chemical context

Monovalent copper halide and pseudohalide coordination compounds have been investigated for several years. First of all, they are of inter­est from a structural point of view, because they show an extremely large structural variability (Kromp & Sheldrick, 1999 ▸; Li *et al.*, 2005 ▸; Peng *et al.*, 2010 ▸), but also because of their luminescence properties (Chesnut *et al.*, 1999 ▸; Lemos *et al.*, 2001 ▸; Näther *et al.*, 2003 ▸; Starosta *et al.*, 2012 ▸; Nitsch *et al.*, 2015 ▸). Two main reasons are responsible for the structural variability. Firstly, the metal cations can be linked by bridging halide anions, which leads to the formation of different Cu*X* substructures such as, for example, mononuclear and dinuclear complexes as well as chains and layers of different topology (Kromp & Sheldrick, 1999 ▸; Näther *et al.*, 2013 ▸). Secondly, for a given copper halide or pseudohalide and a given neutral ligand, compounds with a different ratio between Cu*X* and the organic ligand can be obtained (Näther *et al.*, 2001 ▸, 2002 ▸; Näther & Jess, 2001 ▸). The structural variety can be further enhanced if bridging organic ligands such as pyrazine and its derivatives are used in the synthesis.

In this context, we have reported on a compound with the composition CuNCS(C_6_H_8_N_2_) (C_6_H_8_N_2_ = 2,6-di­methyl­pyrazine) in which the metal cations are fourfold coordinated by one N- and two S-bonded thio­cyanate anions and one 2,6-di­methyl­pyrazine ligand, which coordinate with the N atom that is not adjacent to the two methyl groups to the metal centers (Näther, 2026 ▸). The copper cations are linked by μ-1,1,3(S,S,N)-bridging thio­cyanate anions into corrugated layers and shows a complicated Cu*X* substructure. It is noted that some compounds with copper pseudohalides and 2,6-di­methyl­pyrazine are already reported and they are listed in the *Database survey* section (see below).

Only two compounds are known with copper halides and 2,6-di­methyl­pyrazine. In (CuCl)_2_(2,6-di­methyl­pyrazine), the copper cations are tetra­hedrally coordinated and linked into double chains by μ-1,1,1-bridging chloride anions that condense into layers by bridging 2,6-di­methyl­pyrazine ligands (CSD refcode YEFPOR; Fan *et al.*, 2015*a* ▸). The same double chains are also observed in CuI(2,6-di­methyl­pyrazine) but the 2,6-di­methyl­pyrazine ligand is only terminally coordinated (TONQOE and TONQOE01; Kitada & Ishida, 2014 ▸ and Zhang *et al.*, 2014 ▸). The observation that despite the different ratio between CuX and organic ligand the same Cu*X* substructure is observed in both compounds can be traced back to the fact that 2,6-di­methyl­pyrazine can act as both a terminal and as a ligand because the metal coordination to the N atom that is adjacent to the two methyl groups is sterically hindered. This means that with CuCl, a compound with the composition CuCl(2,6-di­methyl­pyrazine) might exist, in which the 2,6-di­methyl­pyrazine ligand is only terminally coordinated, as is the case in CuI(2,6-di­methyl­pyrazine). If the coligand acts as a bridging ligand, the structure might consists of CuCl single chains that are linked into layers by the coligand as observed in CuCl(pyrazine) [ZOLXED (Moreno *et al.*, 1995 ▸) and ZOLXED01 (Kuhlman *et al.*, 1999 ▸)]. However, as mentioned above, such compounds show an extremely versatile structural behavior, which make structural predictions more difficult.

To prove whether 2,6-di­methyl­pyrazine-rich compounds are available, CuCl was reacted with different amounts of 2,6-di­methyl­pyrazine and because no compounds are known with copper bromide, similar reactions were performed with CuBr. Within these investigations, crystals of one chloride and one bromide compound were obtained and these were characterized by single crystal X-ray diffraction.
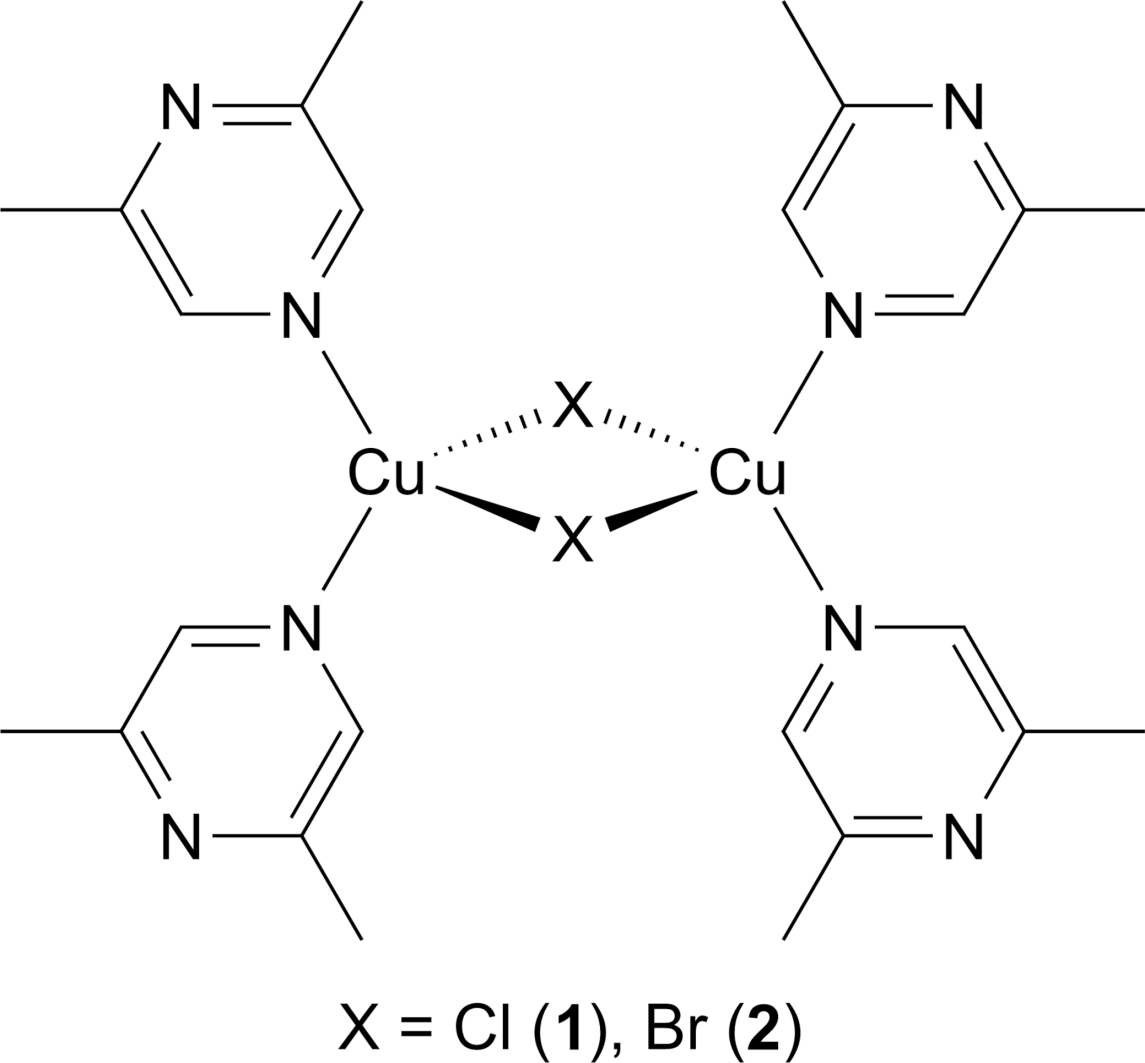


## 2. Structural commentary

[(CuCl)_2_(C_6_H_8_N_2_)_4_] (**1**) and [(CuBr)_2_(C_6_H_8_N_2_)_4_] (**2**) are isotypic. The asymmetric units of both compounds are built up of one crystallographically independent copper cation, one chloride or bromide anion and one 2,6-di­methyl­pyrazine coligand that occupy general positions.

**Figure 1:**
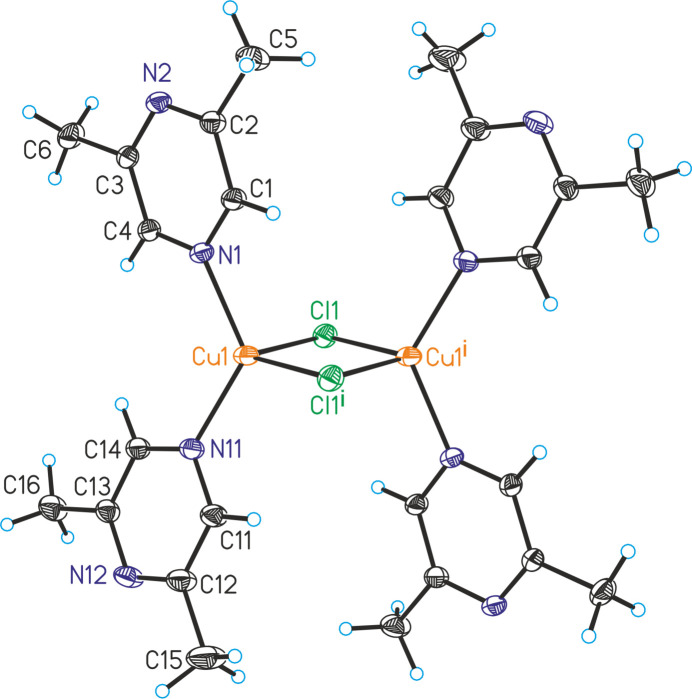
Crystal structure of compound **1** with the atom-labeling scheme. Displacement ellipsoids are drawn at the 50% probability level. Symmetry code: (i) = −*x* + 1, −*y* + 1, −*z* + 1.

The copper(I) cations are fourfold coordinated by two μ-1,1 bridging halide anions and two 2,6-di­methyl­pyrazine ligands that are terminally coordinated by the N atom that is not adjacent to the methyl groups (Figs. 1 ▸ and 2 ▸). Bond lengths and angles show that the tetra­hedra are strongly distorted with the largest values for the N—Cu—N angles, presumably because of steric repulsion between the bulky ligands (Tables 1 ▸ and 2 ▸). Only minor differences in the bonding angles are observed between the chloride and the bromide compounds. Each two copper(I) cations are connected by two μ-1,1 bridging halide anions *via* common edges into discrete dinuclear complexes that are located on centers of inversion (Fig. 1 ▸).

It is noted that this structural motif is very common for this class of compounds and more than 70 structures with chloride anions and *N*-donor coligands are listed in the CSD (Version 5.43, 2026; Groom *et a*l., 2016) using CONQUEST (Bruno *et al.*, 2002 ▸). If this search is limited to pyrazine derivatives, only one hit is found, *viz*. [(CuCl)_2_]_2_(2,3-di­methyl­pyrazine)_6_-2,3-di­methyl­pyrazine solvate (Jess & Näther, 2006*b* ▸), which exhibits a structure very similar to that of the title compound. This compound consists of dinuclear (CuCl)_2_(*L*)_4_ units (*L* = 2,3-di­methyl­pyrazine), but only three of the neutral coligands ligands are terminally coordinated, whereas the fourth ligand acts as a bridging ligand to bind to a second (CuCl)_2_(*L*)_4_ unit. This leads to the formation of tetra­nuclear complexes.

## 3. Supra­molecular features

**Figure 3:**
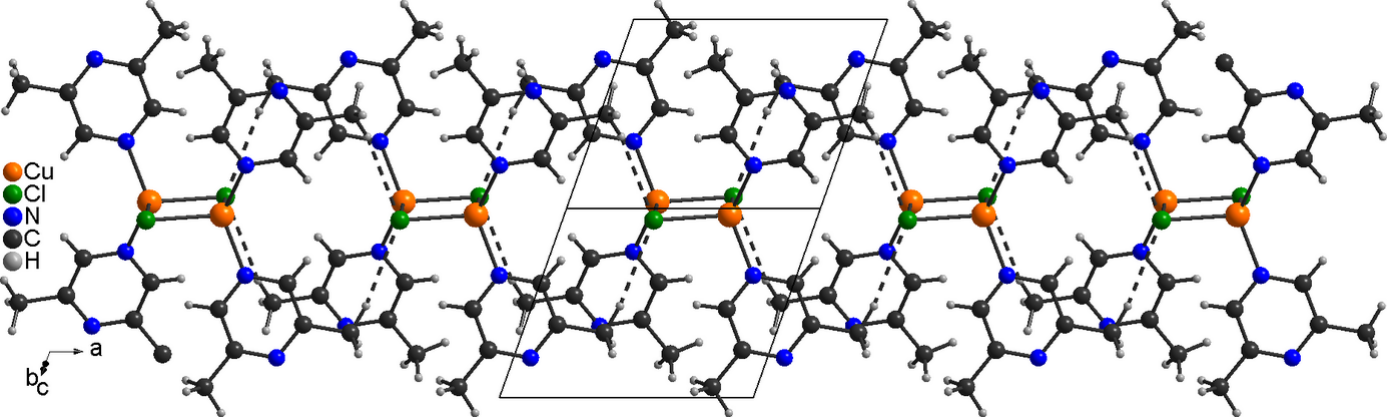
Crystal structure of compound **1** in a view along [01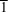]. Inter­molecular C—H⋯N hydrogen bonding is shown as dashed lines. A similar packing arrangement is observed in **2**.

In the extended structure, the dinuclear discrete complexes are linked by centrosymmetric pairs of inter­molecular C—H⋯*X* (*X* = Cl, Br) hydrogen bonding between the halide anions and one of the methyl H atoms (H6*A* and H16*A*, respectively, and H6*B*/H16*B*) into chains, that propagate along the *a*-axis direction (Fig. 3 ▸). There are only minor differences in the H⋯*A* and *D*⋯H distances and the C—H⋯*X* angles are close to linear, indicating that these are relatively strong inter­actions (Tables 3 ▸ and 4 ▸).

**Figure 4:**
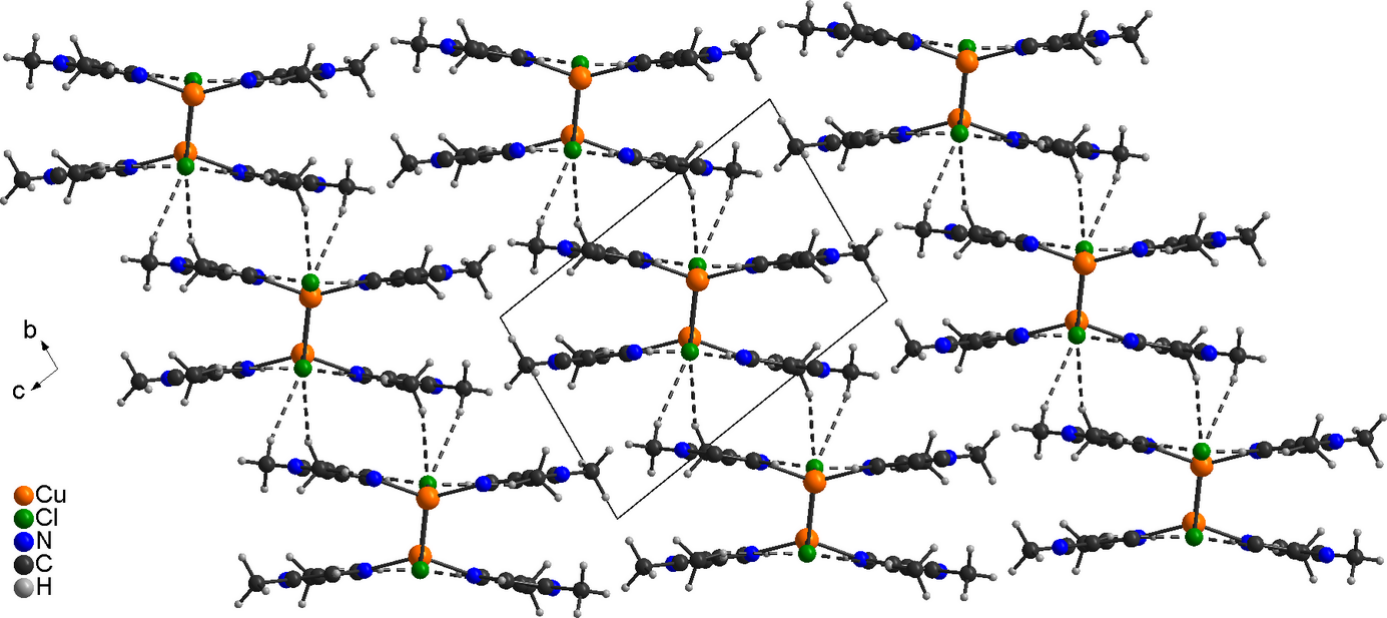
Crystal structure of compound **1** in a view along the crystallographic *a*-axis direction. Inter­molecular C—H⋯N hydrogen bonding is shown as dashed lines. A similar packing arrangement is observed in **2**.

Additional C—H⋯*X* inter­actions are observed between these chains, but for the chloride compound **1** the H⋯*A* and *D*⋯H distances are significantly shorter and the C—H⋯*X* angles are close to linear, which is not the case for the bromide compound **2** (Fig. 4 ▸ and Tables 3 ▸ and 4 ▸). This suggests that the inter­actions between neighbouring chains are stronger in compound **1**.

## 4. Database survey

As mentioned in the *Chemical context* section, some compounds with copper(I) halides or pseudohalides and 2,6-di­methyl­pyrazine are reported in the CSD (Version 5.43, 2025; Groom *et al.*, 2016 ▸) using CONQUEST (Bruno *et al.*, 2002 ▸). These include (CuCl)_2_(2,6-di­methyl­pyrazine) (CSD refcode YEFPOR; Fan *et al.*, 2015*a* ▸) and CuI(2,6-di­methyl­pyrazine) (TONQOE and TONQOE01; Kitada & Ishida, 2014 ▸ and Zhang *et al.*, 2014 ▸), already mentioned above, as well as CuNCS(2,6-di­methyl­pyrazine), which forms CuNCS layers (Näther, 2026 ▸). Two isomers of Cu_2_(CN)_2_(2,6-di­methyl­pyrazine) with copper cyanide show complicated layered CuCN substructures (Fan *et al.*, 2015*b* ▸). Finally, there is a mixed copper(I/II) pseudohalide compound with the composition [Cu_8_^I^Cu_2_^II^(CN)_4_(NCS)_8_(2,6-di­methyl­pyrazine)_7_], which also shows a two-dimensional coordination network (Jess & Näther, 2006*a* ▸).

## 5. Synthesis and crystallization

**General**

Copper(I) chloride, copper(I) bromide and 2,6-di­methyl­pyrazine were purchased from Sigma-Aldrich.

**Synthesis**

In a closed ampoule, 1 mmol of copper(I) halide (CuCl, 99.0 mg; CuBr, 143.5 mg) and 2 mmol of 2,6-di­methyl­pyrazine (216.3 mg) were heated in 2 ml of aceto­nitrile at 413 K for 2 d. After cooling, yellow blocks of compounds **1** and **2** were obtained, which decompose in air.

## 6. Refinement

Crystal data, data collection and structure refinement details are summarized in Table 5 ▸. C—H hydrogen atoms were positioned with idealized geometry (methyl H atoms allowed to rotate but not to tip) and were refined isotropically with *U*_iso_(H) = 1.2*U*_eq_(C) (1.5 for methyl H atoms).

## Supplementary Material

Crystal structure: contains datablock(s) 1, 2. DOI: 10.1107/S2056989026004330/tx2109sup1.cif

Structure factors: contains datablock(s) 1. DOI: 10.1107/S2056989026004330/tx21091sup2.hkl

Structure factors: contains datablock(s) 2. DOI: 10.1107/S2056989026004330/tx21092sup3.hkl

CCDC references: 2549116, 2549115

Additional supporting information:  crystallographic information; 3D view; checkCIF report

## Figures and Tables

**Figure 2:**
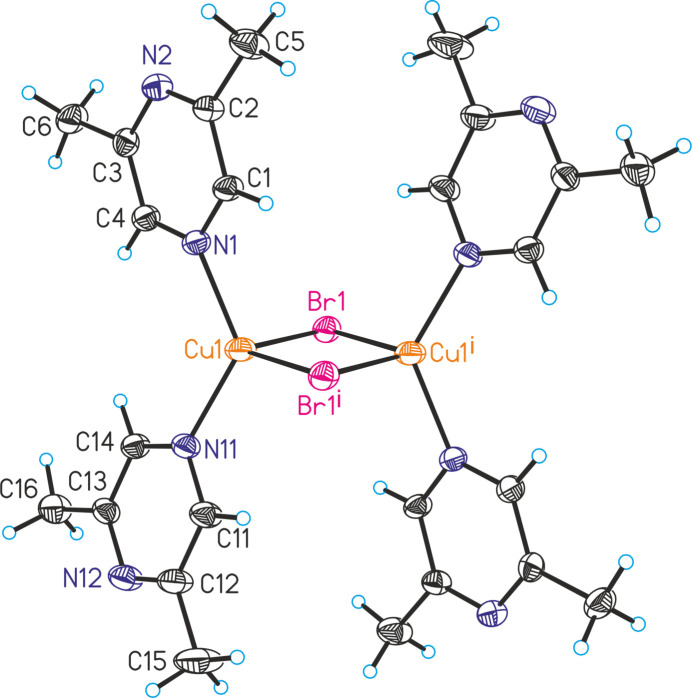
Crystal structure of compound **2** with the atom-labeling scheme. Displacement ellipsoids are drawn at the 50% probability level. Symmetry code: (i) −*x* + 1, −*y* + 1, −*z* + 1.

**Table 1:** Selected geometric parameters (Å, °) for **1**[Chem scheme1]

Cu1—N11	2.0097 (18)	Cu1—Cl1^i^	2.4337 (7)
Cu1—N1	2.0222 (17)	Cu1—Cu1^i^	2.9295 (7)
Cu1—Cl1	2.3893 (7)		
			
N11—Cu1—N1	126.52 (7)	N11—Cu1—Cl1^i^	107.89 (6)
N11—Cu1—Cl1	107.27 (6)	N1—Cu1—Cl1^i^	107.07 (6)
N1—Cu1—Cl1	100.98 (6)	Cl1—Cu1—Cl1^i^	105.20 (2)

Symmetry code: (i) 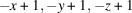.

**Table 2:** Selected geometric parameters (Å, °) for **2**[Chem scheme1]

Cu1—N11	2.016 (3)	Cu1—Br1^i^	2.5513 (6)
Cu1—N1	2.026 (3)	Cu1—Cu1^i^	2.9677 (10)
Cu1—Br1	2.5249 (7)		
			
N11—Cu1—N1	128.12 (13)	N11—Cu1—Br1^i^	107.19 (9)
N11—Cu1—Br1	105.01 (9)	N1—Cu1—Br1^i^	106.55 (9)
N1—Cu1—Br1	100.22 (9)	Br1—Cu1—Br1^i^	108.45 (2)

Symmetry code: (i) 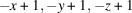.

**Table 3:** Hydrogen-bond geometry (Å, °) for **1**[Chem scheme1]

*D*—H⋯*A*	*D*—H	H⋯*A*	*D*⋯*A*	*D*—H⋯*A*
C6—H6*B*⋯Cl1^ii^	0.98	2.92	3.895 (3)	171
C16—H16*B*⋯Cl1^ii^	0.98	2.96	3.912 (3)	163
C5—H5*C*⋯Cl1^iii^	0.98	2.78	3.716 (3)	159
C6—H6*A*⋯Cl1^iv^	0.98	2.92	3.820 (2)	153

Symmetry codes: (ii) 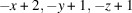; (iii) 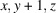; (iv) 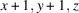.

**Table 4:** Hydrogen-bond geometry (Å, °) for **2**[Chem scheme1]

*D*—H⋯*A*	*D*—H	H⋯*A*	*D*⋯*A*	*D*—H⋯*A*
C6—H6*A*⋯Br1^ii^	0.98	2.98	3.943 (4)	169
C16—H16*A*⋯Br1^ii^	0.98	3.03	3.998 (4)	168
C1—H1⋯Br1^i^	0.95	3.07	3.722 (4)	127
C6—H6*B*⋯Br1^iii^	0.98	3.08	3.920 (4)	144
C11—H11⋯Br1^i^	0.95	3.09	3.739 (4)	127

Symmetry codes: (i) 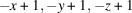; (ii) 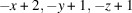; (iii) 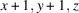.

**Table 5:** Experimental details

	**1**	**2**
Crystal data		
Chemical formula	[Cu_2_Cl_2_(C_6_H_8_N_2_)_4_]	[Cu_2_Br_2_(C_6_H_8_N_2_)_4_]
*M*_r_	630.55	719.47
Crystal system, space group	Triclinic, *P* 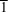	Triclinic, *P* 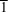
Temperature (K)	170	170
*a*, *b*, *c* (Å)	7.9342 (9), 8.0095 (10), 11.5556 (14)	7.9999 (6), 7.9947 (8), 11.8737 (10)
α, β, γ (°)	95.882 (15), 98.755 (14), 106.416 (14)	97.590 (11), 98.361 (10), 106.618 (10)
*V* (Å^3^)	687.96 (15)	707.77 (12)
*Z*	1	1
Radiation type	Mo *K*α	Mo *K*α
μ (mm^−1^)	1.77	4.35
Crystal size (mm)	0.2 × 0.2 × 0.1	0.15 × 0.12 × 0.11

Data collection		
Diffractometer	Stoe *IPDS*-I	Stoe *IPDS*-I
Absorption correction	Numerical (*X-RED* and *X-SHAPE*; Stoe & Cie, 2002 ▸)	Numerical (*X-RED* and *X-SHAPE*; Stoe & Cie, 2002 ▸)
*T*_min_, *T*_max_	0.714, 0.851	0.551, 0.623
No. of measured, independent and observed [*I* > 2σ(*I*)] reflections	5429, 3171, 2559	7272, 3193, 2332
*R*_int_	0.034	0.040
(sin θ/λ)_max_ (Å^−1^)	0.661	0.660

Refinement		
*R*[*F*^2^ > 2σ(*F*^2^)], *wR*(*F*^2^), *S*	0.033, 0.085, 1.01	0.036, 0.087, 0.96
No. of reflections	3171	3193
No. of parameters	168	164
H-atom treatment	H-atom parameters constrained	H-atom parameters constrained
Δρ_max_, Δρ_min_ (e Å^−3^)	0.35, −0.58	0.59, −0.62

Computer programs: *X-AREA* (Stoe & Cie, 2002 ▸), *SHELXT* (Sheldrick, 2015*a* ▸), *SHELXL* (Sheldrick, 2015*b* ▸), *DIAMOND* (Brandenburg, 1999 ▸), *XP* in *SHELXTL*-PC (Sheldrick, 2008 ▸) and *publCIF* (Westrip, 2010 ▸).

## supplementary crystallographic information

### Di-µ-chlorido-bis[bis(2,6-dimethylpyrazine)copper(I)] (1) Crystal data

**Table:**

[Cu_2_Cl_2_(C_6_H_8_N_2_)_4_]	*Z* = 1
*M**_r_* = 630.55	*F*(000) = 324
Triclinic, *P*1	*D*_x_ = 1.522 Mg m^−^^3^
*a* = 7.9342 (9) Å	Mo *K*α radiation, λ = 0.71073 Å
*b* = 8.0095 (10) Å	Cell parameters from 5965 reflections
*c* = 11.5556 (14) Å	θ = 2.8–28.0°
α = 95.882 (15)°	µ = 1.77 mm^−^^1^
β = 98.755 (14)°	*T* = 170 K
γ = 106.416 (14)°	Block, yellow
*V* = 687.96 (15) Å^3^	0.2 × 0.2 × 0.1 mm

### Di-µ-chlorido-bis[bis(2,6-dimethylpyrazine)copper(I)] (1) Data collection

**Table:**

Stoe IPDS-I diffractometer	2559 reflections with *I* > 2σ(*I*)
Phi scans	*R*_int_ = 0.034
Absorption correction: numerical (X-Red and X-Shape; Stoe & Cie, 2002)	θ_max_ = 28.0°, θ_min_ = 2.7°
*T*_min_ = 0.714, *T*_max_ = 0.851	*h* = −10→10
5429 measured reflections	*k* = −10→10
3171 independent reflections	*l* = −15→14

### Di-µ-chlorido-bis[bis(2,6-dimethylpyrazine)copper(I)] (1) Refinement

**Table:**

Refinement on *F*^2^	Hydrogen site location: inferred from neighbouring sites
Least-squares matrix: full	H-atom parameters constrained
*R*[*F*^2^ > 2σ(*F*^2^)] = 0.033	*w* = 1/[σ^2^(*F*_o_^2^) + (0.0539*P*)^2^] where *P* = (*F*_o_^2^ + 2*F*_c_^2^)/3
*wR*(*F*^2^) = 0.085	(Δ/σ)_max_ < 0.001
*S* = 1.01	Δρ_max_ = 0.35 e Å^−^^3^
3171 reflections	Δρ_min_ = −0.58 e Å^−^^3^
168 parameters	Extinction correction: SHELXL-2016/6 (Sheldrick 2015), Fc^*^=kFc[1+0.001xFc^2^λ^3^/sin(2θ)]^-1/4^
0 restraints	Extinction coefficient: 0.030 (4)

### Di-µ-chlorido-bis[bis(2,6-dimethylpyrazine)copper(I)] (1) Special details

**Table:**

Geometry. All esds (except the esd in the dihedral angle between two l.s. planes) are estimated using the full covariance matrix. The cell esds are taken into account individually in the estimation of esds in distances, angles and torsion angles; correlations between esds in cell parameters are only used when they are defined by crystal symmetry. An approximate (isotropic) treatment of cell esds is used for estimating esds involving l.s. planes.

### Di-µ-chlorido-bis[bis(2,6-dimethylpyrazine)copper(I)] (1) Fractional atomic coordinates and isotropic or equivalent isotropic displacement parameters (Å^2^)

**Table:**

	*x*	*y*	*z*	*U*_iso_*/*U*_eq_	
Cu1	0.65195 (4)	0.58730 (3)	0.44740 (2)	0.02227 (12)	
Cl1	0.64043 (7)	0.36585 (7)	0.57225 (5)	0.02170 (13)	
N1	0.8283 (2)	0.7997 (2)	0.55504 (15)	0.0185 (4)	
C1	0.7716 (3)	0.9041 (3)	0.62846 (19)	0.0197 (4)	
H1	0.647296	0.891650	0.618220	0.024*	
C2	0.8897 (3)	1.0304 (3)	0.71942 (18)	0.0191 (4)	
N2	1.0668 (2)	1.0556 (2)	0.73508 (15)	0.0193 (4)	
C3	1.1249 (3)	0.9534 (3)	0.66057 (19)	0.0179 (4)	
C4	1.0056 (3)	0.8253 (3)	0.57177 (18)	0.0183 (4)	
H4	1.050947	0.754131	0.521620	0.022*	
C5	0.8231 (3)	1.1410 (3)	0.8041 (2)	0.0287 (5)	
H5A	0.924917	1.220955	0.861577	0.043*	
H5B	0.741903	1.064207	0.846063	0.043*	
H5C	0.759116	1.209900	0.759905	0.043*	
C6	1.3226 (3)	0.9814 (3)	0.6766 (2)	0.0258 (5)	
H6A	1.383392	1.102579	0.667025	0.039*	
H6B	1.346472	0.899086	0.617104	0.039*	
H6C	1.367048	0.960824	0.756053	0.039*	
N11	0.6766 (2)	0.4839 (2)	0.28719 (16)	0.0198 (4)	
C11	0.5341 (3)	0.4087 (3)	0.20026 (19)	0.0236 (5)	
H11	0.420381	0.417133	0.211264	0.028*	
C12	0.5480 (3)	0.3180 (3)	0.0938 (2)	0.0261 (5)	
N12	0.7052 (3)	0.3025 (3)	0.07479 (16)	0.0251 (4)	
C13	0.8491 (3)	0.3779 (3)	0.16187 (19)	0.0206 (4)	
C14	0.8341 (3)	0.4689 (3)	0.26692 (19)	0.0206 (4)	
H14	0.938372	0.522019	0.326098	0.025*	
C15	0.3877 (4)	0.2328 (5)	−0.0039 (2)	0.0435 (7)	
H15A	0.420999	0.253686	−0.080588	0.065*	
H15B	0.291496	0.283431	0.007702	0.065*	
H15C	0.346398	0.105722	−0.002240	0.065*	
C16	1.0260 (3)	0.3585 (3)	0.1424 (2)	0.0287 (5)	
H16A	1.012735	0.233860	0.118206	0.043*	
H16B	1.114498	0.403305	0.216083	0.043*	
H16C	1.066046	0.425684	0.080123	0.043*	

### Di-µ-chlorido-bis[bis(2,6-dimethylpyrazine)copper(I)] (1) Atomic displacement parameters (Å^2^)

**Table:**

	*U*^11^	*U*^22^	*U*^33^	*U*^12^	*U*^13^	*U*^23^
Cu1	0.01979 (16)	0.02445 (16)	0.01745 (15)	0.00281 (11)	0.00182 (10)	−0.00620 (10)
Cl1	0.0167 (2)	0.0256 (3)	0.0219 (3)	0.0066 (2)	0.00183 (19)	0.00174 (19)
N1	0.0153 (8)	0.0194 (8)	0.0170 (8)	0.0021 (7)	0.0006 (6)	−0.0023 (7)
C1	0.0133 (10)	0.0242 (10)	0.0194 (10)	0.0052 (8)	0.0019 (8)	−0.0035 (8)
C2	0.0186 (10)	0.0208 (9)	0.0169 (10)	0.0062 (8)	0.0015 (8)	0.0001 (8)
N2	0.0166 (8)	0.0213 (8)	0.0165 (8)	0.0038 (7)	−0.0009 (7)	−0.0007 (7)
C3	0.0139 (10)	0.0203 (9)	0.0183 (10)	0.0040 (8)	0.0024 (7)	0.0016 (8)
C4	0.0156 (10)	0.0189 (9)	0.0193 (10)	0.0044 (8)	0.0037 (8)	−0.0006 (7)
C5	0.0285 (12)	0.0332 (12)	0.0232 (11)	0.0124 (10)	0.0029 (9)	−0.0071 (9)
C6	0.0136 (10)	0.0306 (11)	0.0294 (12)	0.0044 (9)	0.0007 (8)	−0.0013 (9)
N11	0.0183 (9)	0.0235 (9)	0.0156 (8)	0.0056 (7)	0.0019 (7)	−0.0015 (7)
C11	0.0168 (10)	0.0335 (12)	0.0186 (10)	0.0081 (9)	0.0017 (8)	−0.0032 (9)
C12	0.0206 (11)	0.0368 (12)	0.0190 (11)	0.0107 (10)	−0.0007 (8)	−0.0038 (9)
N12	0.0242 (10)	0.0346 (10)	0.0164 (9)	0.0128 (8)	0.0017 (7)	−0.0039 (8)
C13	0.0186 (10)	0.0250 (10)	0.0188 (10)	0.0082 (8)	0.0043 (8)	0.0009 (8)
C14	0.0170 (10)	0.0252 (10)	0.0178 (10)	0.0058 (8)	0.0022 (8)	−0.0013 (8)
C15	0.0259 (13)	0.069 (2)	0.0280 (13)	0.0157 (13)	−0.0059 (10)	−0.0163 (13)
C16	0.0233 (12)	0.0405 (13)	0.0247 (12)	0.0146 (10)	0.0067 (9)	−0.0015 (10)

### Di-µ-chlorido-bis[bis(2,6-dimethylpyrazine)copper(I)] (1) Geometric parameters (Å, º)

**Table:**

Cu1—N11	2.0097 (18)	C6—H6B	0.9800
Cu1—N1	2.0222 (17)	C6—H6C	0.9800
Cu1—Cl1	2.3893 (7)	N11—C11	1.340 (3)
Cu1—Cl1^i^	2.4337 (7)	N11—C14	1.341 (3)
Cu1—Cu1^i^	2.9295 (7)	C11—C12	1.399 (3)
N1—C4	1.343 (3)	C11—H11	0.9500
N1—C1	1.344 (3)	C12—N12	1.336 (3)
C1—C2	1.398 (3)	C12—C15	1.507 (3)
C1—H1	0.9500	N12—C13	1.348 (3)
C2—N2	1.343 (3)	C13—C14	1.389 (3)
C2—C5	1.506 (3)	C13—C16	1.502 (3)
N2—C3	1.346 (3)	C14—H14	0.9500
C3—C4	1.393 (3)	C15—H15A	0.9800
C3—C6	1.500 (3)	C15—H15B	0.9800
C4—H4	0.9500	C15—H15C	0.9800
C5—H5A	0.9800	C16—H16A	0.9800
C5—H5B	0.9800	C16—H16B	0.9800
C5—H5C	0.9800	C16—H16C	0.9800
C6—H6A	0.9800		
			
N11—Cu1—N1	126.52 (7)	C3—C6—H6B	109.5
N11—Cu1—Cl1	107.27 (6)	H6A—C6—H6B	109.5
N1—Cu1—Cl1	100.98 (6)	C3—C6—H6C	109.5
N11—Cu1—Cl1^i^	107.89 (6)	H6A—C6—H6C	109.5
N1—Cu1—Cl1^i^	107.07 (6)	H6B—C6—H6C	109.5
Cl1—Cu1—Cl1^i^	105.20 (2)	C11—N11—C14	116.68 (18)
N11—Cu1—Cu1^i^	119.83 (5)	C11—N11—Cu1	121.77 (15)
N1—Cu1—Cu1^i^	113.52 (5)	C14—N11—Cu1	121.12 (14)
Cl1—Cu1—Cu1^i^	53.291 (18)	N11—C11—C12	121.9 (2)
Cl1^i^—Cu1—Cu1^i^	51.912 (17)	N11—C11—H11	119.1
Cu1—Cl1—Cu1^i^	74.80 (2)	C12—C11—H11	119.1
C4—N1—C1	116.54 (17)	N12—C12—C11	121.0 (2)
C4—N1—Cu1	121.87 (14)	N12—C12—C15	117.0 (2)
C1—N1—Cu1	120.60 (14)	C11—C12—C15	121.9 (2)
N1—C1—C2	122.02 (19)	C12—N12—C13	117.40 (19)
N1—C1—H1	119.0	N12—C13—C14	121.1 (2)
C2—C1—H1	119.0	N12—C13—C16	117.87 (19)
N2—C2—C1	120.97 (19)	C14—C13—C16	121.0 (2)
N2—C2—C5	117.70 (18)	N11—C14—C13	121.9 (2)
C1—C2—C5	121.3 (2)	N11—C14—H14	119.1
C2—N2—C3	117.29 (17)	C13—C14—H14	119.1
N2—C3—C4	121.26 (19)	C12—C15—H15A	109.5
N2—C3—C6	117.78 (18)	C12—C15—H15B	109.5
C4—C3—C6	120.95 (19)	H15A—C15—H15B	109.5
N1—C4—C3	121.89 (19)	C12—C15—H15C	109.5
N1—C4—H4	119.1	H15A—C15—H15C	109.5
C3—C4—H4	119.1	H15B—C15—H15C	109.5
C2—C5—H5A	109.5	C13—C16—H16A	109.5
C2—C5—H5B	109.5	C13—C16—H16B	109.5
H5A—C5—H5B	109.5	H16A—C16—H16B	109.5
C2—C5—H5C	109.5	C13—C16—H16C	109.5
H5A—C5—H5C	109.5	H16A—C16—H16C	109.5
H5B—C5—H5C	109.5	H16B—C16—H16C	109.5
C3—C6—H6A	109.5		
			
C4—N1—C1—C2	−1.4 (3)	C14—N11—C11—C12	0.4 (3)
Cu1—N1—C1—C2	167.41 (16)	Cu1—N11—C11—C12	−172.15 (18)
N1—C1—C2—N2	1.8 (3)	N11—C11—C12—N12	0.2 (4)
N1—C1—C2—C5	−177.4 (2)	N11—C11—C12—C15	179.8 (3)
C1—C2—N2—C3	−0.6 (3)	C11—C12—N12—C13	−0.2 (4)
C5—C2—N2—C3	178.6 (2)	C15—C12—N12—C13	−179.8 (2)
C2—N2—C3—C4	−0.8 (3)	C12—N12—C13—C14	−0.3 (3)
C2—N2—C3—C6	179.5 (2)	C12—N12—C13—C16	178.9 (2)
C1—N1—C4—C3	0.0 (3)	C11—N11—C14—C13	−0.9 (3)
Cu1—N1—C4—C3	−168.65 (16)	Cu1—N11—C14—C13	171.67 (17)
N2—C3—C4—N1	1.1 (3)	N12—C13—C14—N11	0.9 (4)
C6—C3—C4—N1	−179.2 (2)	C16—C13—C14—N11	−178.3 (2)

Symmetry code: (i) −*x*+1, −*y*+1, −*z*+1.

### Di-µ-chlorido-bis[bis(2,6-dimethylpyrazine)copper(I)] (1) Hydrogen-bond geometry (Å, º)

**Table:**

*D*—H···*A*	*D*—H	H···*A*	*D*···*A*	*D*—H···*A*
C6—H6*B*···Cl1^ii^	0.98	2.92	3.895 (3)	171
C16—H16*B*···Cl1^ii^	0.98	2.96	3.912 (3)	163
C5—H5*C*···Cl1^iii^	0.98	2.78	3.716 (3)	159
C6—H6*A*···Cl1^iv^	0.98	2.92	3.820 (2)	153

Symmetry codes: (ii) −*x*+2, −*y*+1, −*z*+1; (iii) *x*, *y*+1, *z*; (iv) *x*+1, *y*+1, *z*.

### Di-µ-bromido-bis[bis(2,6-dimethylpyrazine)dicopper(I)] (2) Crystal data

**Table:**

[Cu_2_Br_2_(C_6_H_8_N_2_)_4_]	*Z* = 1
*M**_r_* = 719.47	*F*(000) = 360
Triclinic, *P*1	*D*_x_ = 1.688 Mg m^−^^3^
*a* = 7.9999 (6) Å	Mo *K*α radiation, λ = 0.71073 Å
*b* = 7.9947 (8) Å	Cell parameters from 6871 reflections
*c* = 11.8737 (10) Å	θ = 2.7–28.0°
α = 97.590 (11)°	µ = 4.35 mm^−^^1^
β = 98.361 (10)°	*T* = 170 K
γ = 106.618 (10)°	Block, yellow
*V* = 707.77 (12) Å^3^	0.15 × 0.12 × 0.11 mm

### Di-µ-bromido-bis[bis(2,6-dimethylpyrazine)dicopper(I)] (2) Data collection

**Table:**

Stoe IPDS-I diffractometer	2332 reflections with *I* > 2σ(*I*)
Phi scans	*R*_int_ = 0.040
Absorption correction: numerical (X-Red and X-Shape; Stoe & Cie, 2002)	θ_max_ = 28.0°, θ_min_ = 2.7°
*T*_min_ = 0.551, *T*_max_ = 0.623	*h* = −9→9
7272 measured reflections	*k* = −10→10
3193 independent reflections	*l* = −15→15

### Di-µ-bromido-bis[bis(2,6-dimethylpyrazine)dicopper(I)] (2) Refinement

**Table:**

Refinement on *F*^2^	Hydrogen site location: inferred from neighbouring sites
Least-squares matrix: full	H-atom parameters constrained
*R*[*F*^2^ > 2σ(*F*^2^)] = 0.036	*w* = 1/[σ^2^(*F*_o_^2^) + (0.0504*P*)^2^] where *P* = (*F*_o_^2^ + 2*F*_c_^2^)/3
*wR*(*F*^2^) = 0.087	(Δ/σ)_max_ < 0.001
*S* = 0.96	Δρ_max_ = 0.59 e Å^−^^3^
3193 reflections	Δρ_min_ = −0.62 e Å^−^^3^
164 parameters	Extinction correction: SHELXL-2016/6 (Sheldrick 2016), Fc^*^=kFc[1+0.001xFc^2^λ^3^/sin(2θ)]^-1/4^
0 restraints	Extinction coefficient: 0.017 (2)

### Di-µ-bromido-bis[bis(2,6-dimethylpyrazine)dicopper(I)] (2) Special details

**Table:**

Geometry. All esds (except the esd in the dihedral angle between two l.s. planes) are estimated using the full covariance matrix. The cell esds are taken into account individually in the estimation of esds in distances, angles and torsion angles; correlations between esds in cell parameters are only used when they are defined by crystal symmetry. An approximate (isotropic) treatment of cell esds is used for estimating esds involving l.s. planes.

### Di-µ-bromido-bis[bis(2,6-dimethylpyrazine)dicopper(I)] (2) Fractional atomic coordinates and isotropic or equivalent isotropic displacement parameters (Å^2^)

**Table:**

	*x*	*y*	*z*	*U*_iso_*/*U*_eq_	
Cu1	0.65082 (6)	0.59508 (6)	0.44939 (4)	0.02835 (15)	
Br1	0.65337 (5)	0.35740 (5)	0.56951 (3)	0.02645 (13)	
N1	0.8239 (4)	0.8099 (4)	0.5599 (2)	0.0244 (6)	
C1	0.7659 (5)	0.9116 (5)	0.6339 (3)	0.0250 (7)	
H1	0.642464	0.898468	0.623456	0.030*	
C2	0.8834 (5)	1.0372 (5)	0.7265 (3)	0.0250 (7)	
N2	1.0577 (4)	1.0627 (4)	0.7434 (2)	0.0258 (6)	
C3	1.1174 (5)	0.9637 (5)	0.6679 (3)	0.0247 (7)	
C4	1.0002 (5)	0.8364 (5)	0.5778 (3)	0.0258 (8)	
H4	1.046083	0.766012	0.527401	0.031*	
C5	0.8129 (6)	1.1438 (6)	0.8114 (3)	0.0356 (9)	
H5A	0.683443	1.111408	0.788259	0.053*	
H5B	0.843087	1.118499	0.888982	0.053*	
H5C	0.866381	1.270626	0.812073	0.053*	
C6	1.3145 (5)	0.9938 (6)	0.6861 (3)	0.0337 (9)	
H6A	1.340434	0.914057	0.625809	0.051*	
H6B	1.375844	1.117285	0.682047	0.051*	
H6C	1.355642	0.969628	0.762323	0.051*	
N11	0.6723 (4)	0.4889 (4)	0.2905 (2)	0.0257 (6)	
C11	0.5296 (5)	0.4163 (5)	0.2047 (3)	0.0307 (8)	
H11	0.418492	0.429462	0.215818	0.037*	
C12	0.5399 (6)	0.3221 (6)	0.0997 (3)	0.0345 (9)	
N12	0.6943 (5)	0.3008 (5)	0.0805 (3)	0.0336 (8)	
C13	0.8378 (5)	0.3746 (5)	0.1648 (3)	0.0263 (8)	
C14	0.8256 (5)	0.4691 (5)	0.2696 (3)	0.0256 (7)	
H14	0.929792	0.520968	0.327670	0.031*	
C15	0.3786 (7)	0.2388 (8)	0.0040 (4)	0.0581 (15)	
H15A	0.275018	0.263779	0.028525	0.087*	
H15B	0.400020	0.288577	−0.065683	0.087*	
H15C	0.356006	0.110124	−0.013101	0.087*	
C16	1.0097 (6)	0.3491 (6)	0.1434 (3)	0.0358 (9)	
H16A	1.103626	0.409358	0.211303	0.054*	
H16B	0.996624	0.222055	0.129384	0.054*	
H16C	1.041353	0.399368	0.075588	0.054*	

### Di-µ-bromido-bis[bis(2,6-dimethylpyrazine)dicopper(I)] (2) Atomic displacement parameters (Å^2^)

**Table:**

	*U*^11^	*U*^22^	*U*^33^	*U*^12^	*U*^13^	*U*^23^
Cu1	0.0243 (3)	0.0316 (3)	0.0236 (2)	0.0064 (2)	0.00211 (18)	−0.00626 (18)
Br1	0.0199 (2)	0.0311 (2)	0.02680 (19)	0.00841 (14)	0.00289 (13)	0.00052 (14)
N1	0.0198 (17)	0.0263 (15)	0.0229 (13)	0.0045 (12)	0.0026 (11)	−0.0023 (11)
C1	0.0162 (19)	0.0306 (19)	0.0244 (17)	0.0068 (14)	0.0006 (13)	−0.0035 (14)
C2	0.027 (2)	0.0250 (17)	0.0217 (16)	0.0087 (14)	0.0025 (13)	−0.0005 (13)
N2	0.0248 (18)	0.0262 (15)	0.0243 (14)	0.0078 (12)	0.0010 (12)	0.0016 (12)
C3	0.0189 (19)	0.0288 (18)	0.0266 (17)	0.0073 (14)	0.0054 (13)	0.0050 (14)
C4	0.021 (2)	0.0278 (18)	0.0259 (17)	0.0062 (15)	0.0053 (13)	−0.0016 (13)
C5	0.032 (2)	0.046 (2)	0.0289 (18)	0.0186 (18)	0.0032 (15)	−0.0052 (17)
C6	0.019 (2)	0.040 (2)	0.036 (2)	0.0058 (16)	0.0019 (15)	−0.0017 (17)
N11	0.0222 (18)	0.0326 (16)	0.0202 (13)	0.0081 (13)	0.0042 (11)	−0.0017 (11)
C11	0.022 (2)	0.044 (2)	0.0232 (17)	0.0115 (16)	0.0034 (14)	−0.0044 (15)
C12	0.026 (2)	0.048 (2)	0.0235 (17)	0.0113 (18)	0.0005 (14)	−0.0080 (16)
N12	0.030 (2)	0.045 (2)	0.0228 (15)	0.0139 (15)	0.0030 (12)	−0.0044 (13)
C13	0.024 (2)	0.0335 (19)	0.0218 (16)	0.0094 (15)	0.0062 (13)	0.0018 (14)
C14	0.021 (2)	0.0291 (18)	0.0240 (16)	0.0075 (14)	0.0022 (13)	−0.0012 (14)
C15	0.030 (3)	0.097 (4)	0.033 (2)	0.022 (3)	−0.0070 (18)	−0.026 (2)
C16	0.026 (2)	0.048 (2)	0.033 (2)	0.0153 (18)	0.0072 (15)	−0.0029 (17)

### Di-µ-bromido-bis[bis(2,6-dimethylpyrazine)dicopper(I)] (2) Geometric parameters (Å, º)

**Table:**

Cu1—N11	2.016 (3)	C6—H6B	0.9800
Cu1—N1	2.026 (3)	C6—H6C	0.9800
Cu1—Br1	2.5249 (7)	N11—C14	1.334 (5)
Cu1—Br1^i^	2.5513 (6)	N11—C11	1.344 (5)
Cu1—Cu1^i^	2.9677 (10)	C11—C12	1.394 (5)
N1—C1	1.340 (5)	C11—H11	0.9500
N1—C4	1.345 (5)	C12—N12	1.342 (6)
C1—C2	1.403 (5)	C12—C15	1.511 (6)
C1—H1	0.9500	N12—C13	1.338 (5)
C2—N2	1.331 (5)	C13—C14	1.400 (5)
C2—C5	1.507 (5)	C13—C16	1.498 (6)
N2—C3	1.346 (5)	C14—H14	0.9500
C3—C4	1.391 (5)	C15—H15A	0.9800
C3—C6	1.504 (6)	C15—H15B	0.9800
C4—H4	0.9500	C15—H15C	0.9800
C5—H5A	0.9800	C16—H16A	0.9800
C5—H5B	0.9800	C16—H16B	0.9800
C5—H5C	0.9800	C16—H16C	0.9800
C6—H6A	0.9800		
			
N11—Cu1—N1	128.12 (13)	C3—C6—H6B	109.5
N11—Cu1—Br1	105.01 (9)	H6A—C6—H6B	109.5
N1—Cu1—Br1	100.22 (9)	C3—C6—H6C	109.5
N11—Cu1—Br1^i^	107.19 (9)	H6A—C6—H6C	109.5
N1—Cu1—Br1^i^	106.55 (9)	H6B—C6—H6C	109.5
Br1—Cu1—Br1^i^	108.45 (2)	C14—N11—C11	116.6 (3)
N11—Cu1—Cu1^i^	118.32 (9)	C14—N11—Cu1	121.3 (2)
N1—Cu1—Cu1^i^	113.31 (9)	C11—N11—Cu1	121.7 (3)
Br1—Cu1—Cu1^i^	54.639 (19)	N11—C11—C12	121.9 (4)
Br1^i^—Cu1—Cu1^i^	53.811 (18)	N11—C11—H11	119.1
Cu1—Br1—Cu1^i^	71.55 (2)	C12—C11—H11	119.1
C1—N1—C4	116.7 (3)	N12—C12—C11	120.9 (4)
C1—N1—Cu1	120.7 (3)	N12—C12—C15	117.4 (3)
C4—N1—Cu1	121.5 (2)	C11—C12—C15	121.7 (4)
N1—C1—C2	121.4 (3)	C13—N12—C12	117.7 (3)
N1—C1—H1	119.3	N12—C13—C14	120.7 (4)
C2—C1—H1	119.3	N12—C13—C16	117.6 (3)
N2—C2—C1	121.5 (3)	C14—C13—C16	121.7 (3)
N2—C2—C5	118.4 (3)	N11—C14—C13	122.2 (3)
C1—C2—C5	120.1 (4)	N11—C14—H14	118.9
C2—N2—C3	117.4 (3)	C13—C14—H14	118.9
N2—C3—C4	121.0 (3)	C12—C15—H15A	109.5
N2—C3—C6	117.5 (3)	C12—C15—H15B	109.5
C4—C3—C6	121.5 (3)	H15A—C15—H15B	109.5
N1—C4—C3	122.0 (3)	C12—C15—H15C	109.5
N1—C4—H4	119.0	H15A—C15—H15C	109.5
C3—C4—H4	119.0	H15B—C15—H15C	109.5
C2—C5—H5A	109.5	C13—C16—H16A	109.5
C2—C5—H5B	109.5	C13—C16—H16B	109.5
H5A—C5—H5B	109.5	H16A—C16—H16B	109.5
C2—C5—H5C	109.5	C13—C16—H16C	109.5
H5A—C5—H5C	109.5	H16A—C16—H16C	109.5
H5B—C5—H5C	109.5	H16B—C16—H16C	109.5
C3—C6—H6A	109.5		
			
C4—N1—C1—C2	−1.0 (5)	C14—N11—C11—C12	1.2 (6)
Cu1—N1—C1—C2	166.5 (3)	Cu1—N11—C11—C12	−171.4 (3)
N1—C1—C2—N2	1.2 (6)	N11—C11—C12—N12	−0.1 (7)
N1—C1—C2—C5	−177.2 (3)	N11—C11—C12—C15	179.3 (4)
C1—C2—N2—C3	0.2 (5)	C11—C12—N12—C13	−0.8 (6)
C5—C2—N2—C3	178.7 (3)	C15—C12—N12—C13	179.8 (4)
C2—N2—C3—C4	−1.8 (5)	C12—N12—C13—C14	0.6 (6)
C2—N2—C3—C6	179.3 (3)	C12—N12—C13—C16	179.5 (4)
C1—N1—C4—C3	−0.5 (5)	C11—N11—C14—C13	−1.4 (5)
Cu1—N1—C4—C3	−168.0 (3)	Cu1—N11—C14—C13	171.2 (3)
N2—C3—C4—N1	2.0 (6)	N12—C13—C14—N11	0.5 (6)
C6—C3—C4—N1	−179.1 (3)	C16—C13—C14—N11	−178.3 (4)

Symmetry code: (i) −*x*+1, −*y*+1, −*z*+1.

### Di-µ-bromido-bis[bis(2,6-dimethylpyrazine)dicopper(I)] (2) Hydrogen-bond geometry (Å, º)

**Table:**

*D*—H···*A*	*D*—H	H···*A*	*D*···*A*	*D*—H···*A*
C6—H6*A*···Br1^ii^	0.98	2.98	3.943 (4)	169
C16—H16*A*···Br1^ii^	0.98	3.03	3.998 (4)	168
C1—H1···Br1^i^	0.95	3.07	3.722 (4)	127
C6—H6*B*···Br1^iii^	0.98	3.08	3.920 (4)	144
C11—H11···Br1^i^	0.95	3.09	3.739 (4)	127

Symmetry codes: (i) −*x*+1, −*y*+1, −*z*+1; (ii) −*x*+2, −*y*+1, −*z*+1; (iii) *x*+1, *y*+1, *z*.
